# Enhancing maize growth through the synergistic impact of potassium enrich biochar and spermidine

**DOI:** 10.1186/s12870-024-04722-4

**Published:** 2024-01-09

**Authors:** Shoucheng Huang, Ping Huang, Sajid Masood, Muhammad Mazhar Iqbal, Tayyaba Naz, Subhan Danish, Mohammad Javed Ansari, Saleh H. Salmen

**Affiliations:** 1 College of Life and Health Science, Anhui Science and Technology University, Fengyang, 233100 China; 2 College of Chemistry and Materials Engineering, Anhui Science and Technology University, Bengbu, 233000 China; 3 Department of Soil Science, Faculty of Agricultural Sciences and Technology, Bahauddin Zakariya University, Multan, Punjab Pakistan; 4 Department of Soil and Environmental Sciences, College of Agriculture, University of Sargodha, Sargodha, 40100 Pakistan; 5 Saline Agriculture Research Centre, Institute of Soil and Environmental Sciences, University of Agriculture Faisalabad, Faisalabad, 38400 Pakistan; 6 Department of Botany, Hindu College Moradabad (MJP Rohilkhand University Bareilly), Moradabad, 244001 India; 7 Department of Botany and Microbiology, College of Science, King Saud University, PO Box -2455, Riyadh, 11451 Saudi Arabia

**Keywords:** Chlorophyll content, Growth attributes, Nutrients, Potassium-rich biochar, Spermidine

## Abstract

Maize cultivated for dry grain covers approximately 197 million hectares globally, securing its position as the second most widely grown crop worldwide after wheat. Although spermidine and biochar individually showed positive impacts on maize production in existing literature, their combined effects on maize growth, physiology, nutrient uptake remain unclear and require further in-depth investigation. That’s why a pot experiment was conducted on maize with spermidine and potassium enriched biochar (KBC) as treatments in Multan, Pakistan, during the year 2022. Four levels of spermidine (0, 0.15, 0.30, and 0.45mM) and two levels of potassium KBC (0 and 0.50%) were applied in completely randomized design (CRD). Results showed that 0.45 mM spermidine under 0.50% KBC caused significant enhancement in maize shoot length (11.30%), shoot fresh weight (25.78%), shoot dry weight (17.45%), root length (27.95%), root fresh weight (26.80%), and root dry weight (20.86%) over control. A significant increase in maize chlorophyll a (50.00%), chlorophyll b (40.40%), total chlorophyll (47.00%), photosynthetic rate (34.91%), transpiration rate (6.51%), and stomatal conductance (15.99%) compared to control under 0.50%KBC validate the potential of 0.45 mM spermidine. An increase in N, P, and K concentration in the root and shoot while decrease in electrolyte leakage and antioxidants also confirmed that the 0.45 mM spermidine performed more effectively with 0.50%KBC. In conclusion, 0.45 mM spermidine with 0.50%KBC is recommended for enhancing maize growth.

## Introduction

Maize, cultivated for its dry grain, extends across about 197 million hectares worldwide, firmly establishing itself as the second most extensively grown crop globally, following only wheat [1]. Maize, an essential staple cereal crop [2–4], supports the ever-increasing human population directly as foodstuff or indirectly as a feed for livestock [5]. It is the third most important crop after rice and wheat [6]. However, poor soil organic matter [7–9] and low beneficial microbes [10, 11] proliferations are one of the growths limiting factor for maize.

Biochar is a multi-functional carbon-rich material, a relative form of charcoal commonly produced from a wide range of biomass, including woody material, livestock material, crop straw, and organic waste [9, 12–14]. Its properties mainly depend upon the type of biomass pyrolysis temperature and residence time [15]. It is a promising ameliorant that can improve crop growth by modulating soil conditions due to its unique characteristics, such as large surface area, abundant oxygen-containing functional groups, rich pore structure, and high cation exchange capacity (CEC) [16, 17].

Spermidine belongs to the polyamines group; polyamines are small aliphatic amines commonly present in plants, animals, and microorganisms [18]. Literature reports polyamines act as a second messenger and play a key role in plant growth, germination, enzyme activation, and stress tolerance [19]. Considerable research reports exhibit that exogenous source polyamines mitigate the adverse effects of stress by regulating ROS production and activating tolerance mechanisms; spermidine is widely used [20].

However, limited studies are present on the interactive effect of potassium-rich biochar and spermidine in enhancing the growth and yield of crop plants. Therefore, the present study aims to investigate the synergistic impact of potassium-rich biochar and spermidine on the growth on nutrients uptake, growth, and physiology of maize crop.

## Materials and methods

### Experimental site

**Fig. 1:**
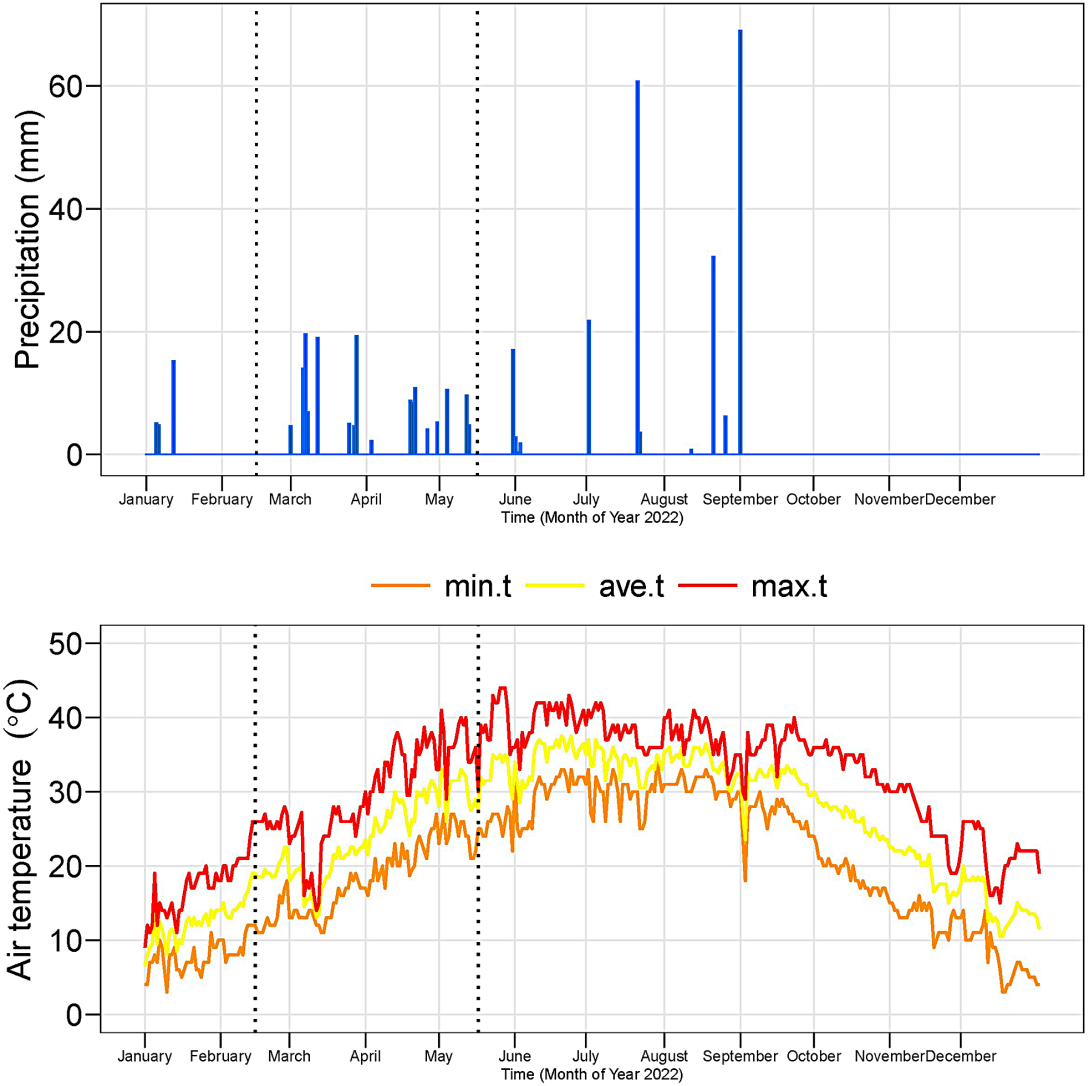
The minimum (min.t), maximum (max.t), and average temperature (ave.t) on the experiment site during year 2022)

The pot experiment was conducted in Faculty of Agricultural Sciences and Technology, Bahauddin Zakariya University, Multan, Pakistan, during spring 2022. The climatic data of the experimental site is provided in Fig. 1.

A total of 10 individual soil samples were collected and combined to create a composite sample for the initial soil characterization. The soil texture analysis used a hydrometer with the USDA textural triangle [21]. A pre-calibrated pH meter and electrical conductivity (EC) meter were utilized to determine soil pH and EC. This involved preparing soil: deionized water mixtures in ratios of 1:1 and 1:10, respectively [22, 23]. The soil organic matter content analysis was conducted following the potassium dichromate methodology. The final values were determined through titration with ferrous ammonium sulfate [24]. Total soil nitrogen content was assessed using Kjeldahl’s distillation method [25].

Additionally, available phosphorus and potassium were evaluated using Olsen and ammonium acetate extracting reagents, respectively [26, 27]. The final values for phosphorus were obtained by measuring the absorbance at 880 nm wavelength using a spectrophotometer, while potassium levels were determined using a flame photometer.

### Preparation and characterization of biochar

Local manufactured Kiln was used to prepare biochar. The temperature was maintained at 350 ^o^C for 3 h. Cotton sticks were used as the primary raw material to produce biochar, which was then subjected to pyrolysis at a temperature of 440 °C. After cooling, the biochar was grinded and passed from 2 mm sieve. Finally, biochar was stored in powder form for upcoming uses. For characterization [21], a 1:10 mixture of biochar and distilled water was made, and samples were then examined to determine the pH [28] and electrical conductivity (EC) [29]. The biochar samples were digested and then distilled using the Kjeldahl distillation apparatus to determine nitrogen (N) [30]. Using a combination of HNO_3_ and HClO_4_ (in 2:1 ratio), digestion of biochar was performed. After that digested material was used for analysis of phosphorus (P) and potassium (K^+^) on spectrophotometer (ammonium vanadate-ammonium molybdate) and flamephotometer [27, 31]. Pre-experimental soil characteristics are provided in Table 1.

**Table 1:** Pre-experimental soil, irrigation water and biochar characteristics

Soil		Water		Biochar	
Sand (%)	50	pH	7.02	pH	8.06
Silt (%)	40	TDS (ppm)	313	EC (mS/cm)	4.56
Clay (%)	10	CO_3_^− 2^ (meq./L)	0.00	Ash content (%)	12.40
Texture	Loam	HCO_3_^− 1^ (meq./L)	5.18	Volatile matter (%)	16.50
pH*s*	8.01	Cl^− 1^ (meq./L)	0.05	Fixed Carbon (%)	71.10
EC*e* (dS/m)	2.03	Na^+ 1^ (meq./L)	2.01	Total N (%)	0.025
Organic Matter (%)	0.40	Ca^+ 2^+Mg^+ 2^ (meq./L)	3.11	Total P (%)	0.61
Available P (µg/g)	5.31	SAR	1.61	Total K (%)	0.33
Extractable K (µg/g)	189	RSC (meq./L)	2.07	Total Ca (%)	1.62

### Potassium enriches biochar

To make the potassium enriched biochar (KBC), the biochar was soaked in a potassium-rich solution (25% K) which was made using potassium sulfate. After 72 h incubation, the material was dried in an oven at 65 °C for 72 h. The final powder was sieved again from a 2 mm sieve and used for further experiments.

### Spermidine

Spermidine was purchased from a certified dealer of SIGMA in the vicinity of Multan. The product with identification number S0266, bearing batch number BCCK9651 and branded as SIGMA, corresponds to CAS Number 124-20-9 with the chemical formula C7H19N3 and a formula weight of 145.25 g/mol.

### Treatments plan

The KBC was mixed in soil during pot filling. There were 2 levels of KBC i.e., 0 and 0.5% w/w. Four levels of spermidine were applied as foliar i.e., 0, 0.15, 0.30 and 0.45mM. The treatments include control (0mM spermidine + 0KBC), 0.15mM spermidine, 0.30mM spermidine, 0.45mM spermidine, 0.50% potassium enriched biochar (0.5KBC), 0.15mM spermidine + 0.50KBC, 0.30mM spermidine + 0.50KBC and 0.45mM spermidine + 0.50KBC. All the treatments were applied following completely randomized design (CRD). A total of 3 foliar applications of spermidine were done at early growth (1st = 10 days after sowing), 2nd = at 25 days of sowing and 3rd at 45 days after sowing.

### Fertilizer

To meet the nutritional needs of maize, nitrogen (N), phosphorus (P) and potassium (K) were added in soil at the rate of 200 (1.0 g/10kg soil), 150 (0.75/10kg soil), and 100 (0.50/10 kg soil) kg ha^− 1^, respectively. Urea was employed as the N source, and single superphosphate was utilized for P and K based on the specified requirements.

### Maize seeds sterilization

The Cimmyt-Pak (variety of maize) seeds used in this experiment were purchased from a licensed seed seller in Multan, Punjab, Pakistan. Before the sowing phase, a surface sterilization procedure was followed. This entailed subjecting the seeds to a 5% sodium hypochlorite solution, succeeded by three consecutive rinses employing 95% ethanol. Subsequently, the seeds underwent three additional rinses with sterilized deionized water to eliminate any remnants of the sterilizing agents [32].

### Pots dimensions and sowing

The experiment utilized plastic pots with a diameter of 10 inches and depth of 16 inches. Each pot containing 10 kg of soil was initially sown with 10 seeds. Following germination, a careful thinning process was carried out, resulting in maintenance of 2 healthy seedlings in each pot.

### Irrigation

Throughout the experiment the soil moisture was maintained at 70% field capacity by using soil moisture meter (Cubilan 4 in 1 Soil Moisture Meter).

### Data collection and harvesting

After 60 days following the sowing, the samples were collected to collect the necessary data. Data collection involved measuring the shoot length and root length using a measuring scale. Additionally, leaves were gathered from each replication, and the fresh weight of leaves was recorded. Subsequently, the fresh weights of the shoots and roots were measured upon harvest. To ascertain the dry weights of the roots and shoots, samples were subjected to drying in an oven set at 65 ± 3 °C for 72 h, ensuring that a consistent weight was achieved.

### Chlorophyll contents and carotenoids

We followed Arnon procedure to measure the chlorophyll a, b, & total chlorophyll in maize leaves [33]. We evaluated absorbance at three different wavelengths throughout the purification process: 663 nm for chlorophyll a, 645 nm for chlorophyll b, and 480 nm for carotenoids.

$$\text{C}\text{h}\text{l}\text{o}\text{r}\text{o}\text{p}\text{h}\text{y}\text{l}\text{l} \text{ a} \left(\frac{\text{m}\text{g}}{\text{g}}\right)=\frac{\left(12.7 \times \text{A}663\right) - \left(2.69 \times \text{A}645\right)\times \text{V}}{1000 \times \text{W}}$$

$$\text{C}\text{h}\text{l}\text{o}\text{r}\text{o}\text{p}\text{h}\text{y}\text{l}\text{l} \text{ b} \left(\frac{\text{m}\text{g}}{\text{g}}\right)=\frac{\left(22.9 \times \text{A}645\right) - \left(4.68 \times \text{A}645\right)\times \text{V}}{1000 \times \text{W}}$$

$$\text{T}\text{o}\text{t}\text{a}\text{l } \text{C}\text{h}\text{l}\text{o}\text{r}\text{o}\text{p}\text{h}\text{y}\text{l}\text{l} \left(\frac{\text{m}\text{g}}{\text{g}}\right)= 20.2\left(\text{O}\text{D} 645\right)+8.02\left(\text{O}\text{D} 663\right)\times \text{V}/1000 \left(\text{W}\right)$$

$$\text{C}\text{a}\text{r}\text{o}\text{t}\text{e}\text{n}\text{o}\text{i}\text{d}\text{s} \left(\frac{\text{m}\text{g}}{\text{g}}\right)=\text{O}\text{D}480+0.114 \left(\text{O}\text{D}663\right)-0.638 \left(\text{O}\text{D}645\right)$$

### Gas exchange attributes

An infrared gas analyzer (CID, Inc. USA-produced CI-340) was used to measure the stomatal conductance, photosynthetic rate, and gas exchange attributes in maize leaves. The analysis were done in a sunny day between 10:30 and 11:30 AM [34].

### Antioxidants

To prepare the enzyme extract for antioxidants, 0.5 g of leaves material was ground with 10 cm^3^ of chilled buffer in a pre-chilled mortar and pestle. For superoxide dismutase (SOD) and catalase (CAT), the extraction medium consisted of 0.1 M potassium phosphate buffer (pH 7.5) containing 0.5 mM EDTA. In the case of ascorbate peroxidase (APx), the extraction utilized 0.1 M phosphate buffer (pH 7) containing 1 mM ascorbic acid. The resulting slurry was filtered through cheesecloth and the filtrate was centrifuged for 15 min, yielding the supernatant referred to as the enzyme extract. The inhibition of nitro blue tetrazolium (NBT) decrease in the presence of riboflavin was studied to ascertain SOD activity. The resulting mixture, which included riboflavin, phosphate buffer, NBT, and enzyme extract, was lighted while the change in absorbance at 560 nm was observed [35]. Monitoring the oxidation of a suitable substrate, such as guaiacol or o-dianisidine, was used to measure peroxidase activity. At a specific wavelength, the rise in absorbance carried on by substrate oxidation was observed [36]. Monitoring the breakdown of hydrogen peroxide (H_2_O_2_) by the catalase enzyme was used to measure its activity. It was tested how much H_2_O_2_ breakdown reduced the absorbance at 240 nm [37]. Ascorbate oxidation in the presence of H_2_O_2_ is observed for APX activity [38]. Over time, it was possible to detect a reduction in absorbance at a particular wavelength. The amount of MDA, a marker of lipid peroxidation, was measured by forming a colored complex by reacting the sample extract with thiobarbituric acid (TBA). The complex’s absorbance was determined, and the MDA content was determined.

### Electrolyte leakage

Before the analysis, the leaves were washed with deionized water to remove any exterior contaminants. Then, using a steel cylinder with a 1 cm diameter, we obtained leaves sections of uniform size & weight, each weighing around one gram. After that, each leaf’s part was put into a different test tube with 20 ml of deionized water. To encourage the diffusion of electrolytes from the leaf’s tissues into the surrounding water, the test tubes were incubated at 25 °C for 24 h. We then used a pre-calibrated EC meter to determine the water solution’s baseline electrical conductivity (EC1). Applying the procedure described by [39], the test tubes were then heated in a water bath at 120 °C for 20 min to measure the second electrical conductivity (EC2).

$$\text{Electrolyte}\,\text{Leakage}\,(\%) = \left( \frac{\text{EC}1}{\text{EC}2} \right) \times 100 $$

### Shoot and root N, P, and K

A customized micro-kjeldahl technique was employed to assess the nitrogen levels [40]. A flame photometer built into continuous-flow systems (the Italian microflow automated continuous-flow analyzer III) was used to measure the potassium levels. A spectrophotometer was simultaneously used to detect the amount of phosphorus using the yellow color technique at 420 nm [41].

### Statistical analysis

The assessment of treatment significance used a two-way ANOVA using OriginPro Program. The Tukey test was used for the paired comparison for the treatment with a *p*-value of 0.05. Using the OriginPro program, convex hull cluster plots, hierarchical cluster plots, and Pearson correlation were carried out.

## Results

### Shoot length, fresh and dry weight

**Fig. 2:**
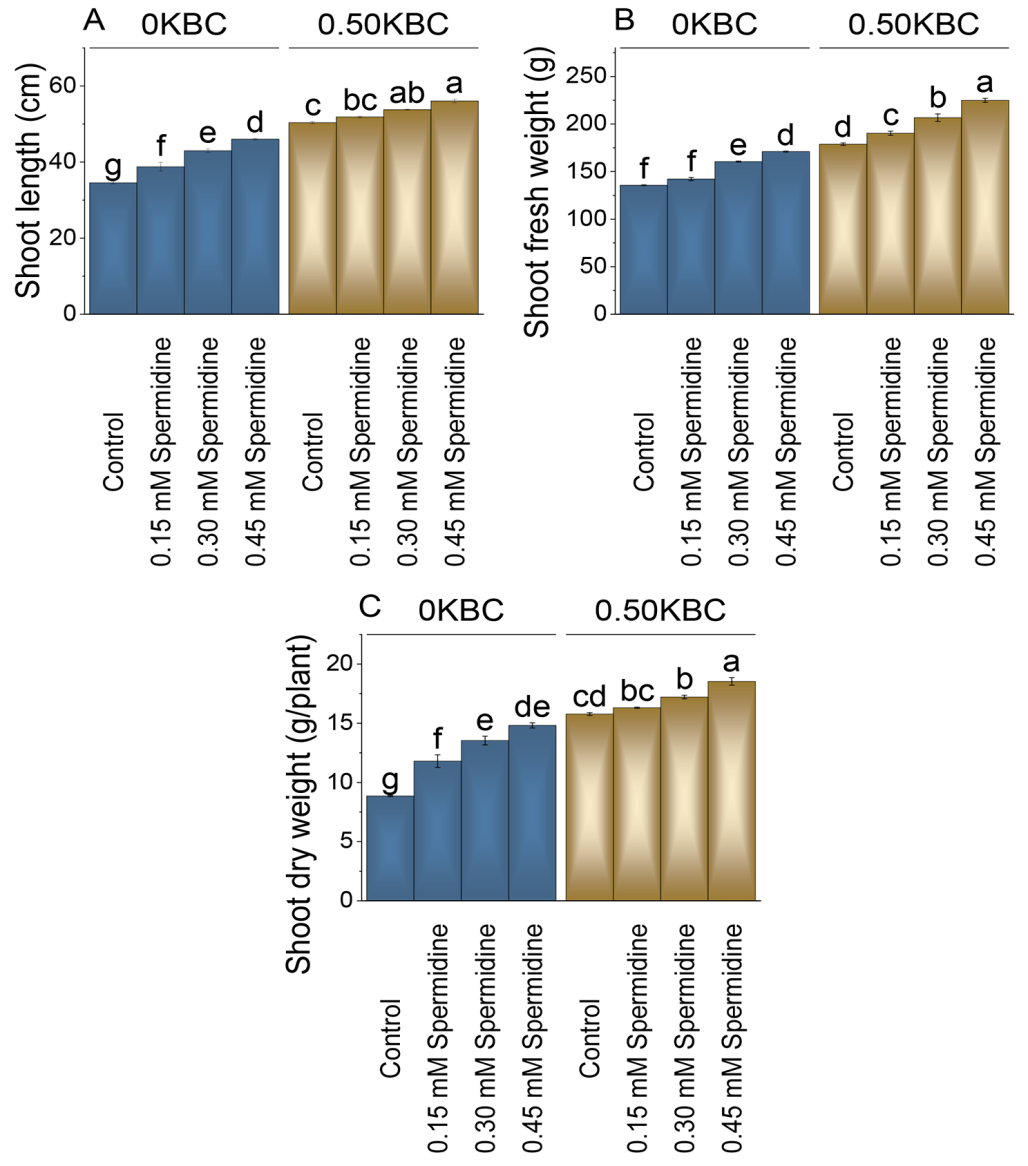
Effect of treatments on shoot length (**A**), shoot fresh weight (**B**), and shoot dry weight (**C**) of maize cultivated under 0KBC and 0.50KBC. The means of four replications are represented by the bars, with standard error. Tukey test results that used different letters on bars indicated significant differences at p ≤ 0.05

Shoot length increased by 12.19%, 24.40%, and 33.23% with 0.15, 0.30, and 0.45 mM spermidine with 0 KBC compared to the control. In the case of the 0.50KBC treatment, the control group had a shoot length of 50.36 cm. With 0.15 mM spermidine with 0.50KBC, there was a 2.95% increase in shoot length, and treatment with 0.30 mM spermidine resulted in a 6.78% increase. The highest increase of 11.30% was observed with 0.45 mM spermidine with 0.50KBC in shoot length compared to the control (Fig. 2A).

The 0.45 mM spermidine treatment with OKBC showed a 26.11% rise in shoot fresh weight contrasted to the control treatment. In the 0.50KBC treatment group, the control had a shoot fresh weight of 178.80 cm. When 0.15 mM spermidine was added with 0.50KBC, there was a 6.43% increase in shoot fresh weight, and the application of 0.30 mM spermidine resulted in a significant 15.62% increase. The 0.45 mM spermidine treatment with 0.50KBC showed a remarkable 25.78% increase in shoot fresh weight compared to the control (Fig. 2B).

In the absence of KBC (0KBC), applying 0.15 mM spermidine led to a 33.24% increase in shoot dry weight compared to the control. This effect intensified with higher spermidine concentrations, resulting in a 52.89% increase at 0.30 mM and a remarkable 67.33% increase at 0.45 mM at 0KNC over the control treatment. At the 0.50 KBC level, the control exhibited a baseline value, with subsequent increases in shoot dry weight as spermidine concentrations rose. These increases were more significant with 0.50 KBC, resulting in a 3.36% rise at 0.15 mM spermidine, a 9.10% increase at 0.30 mM spermidine, and a more substantial 17.45% increase at 0.45 mM spermidine related to the control (Fig. 2C).

### Root length, fresh and dry weight

**Fig. 3:**
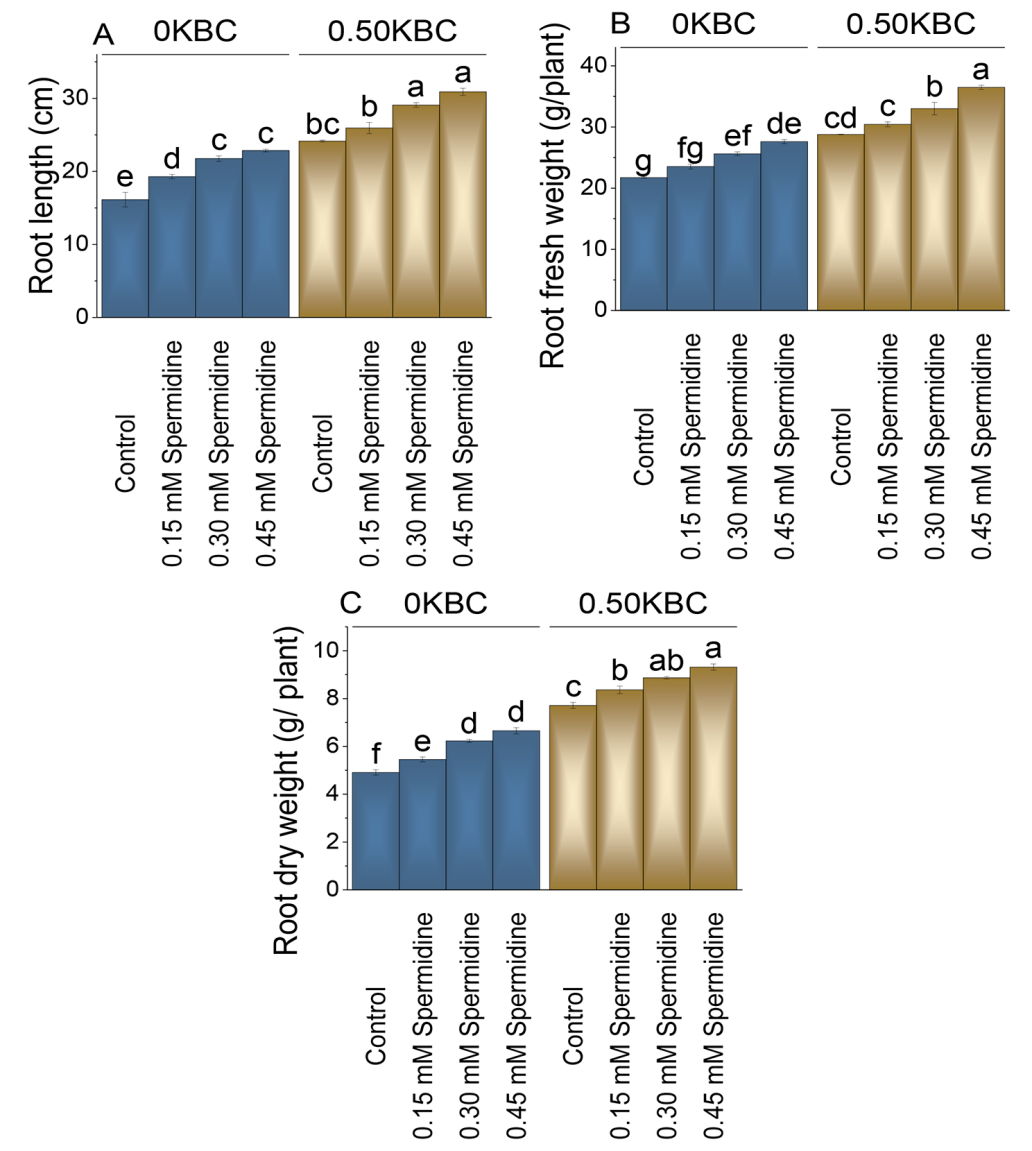
Effect of treatments on root length (**A**), root fresh weight (**B**), and root dry weight (**C**) of maize cultivated under 0KBC and 0.50KBC. The means of four replications are represented by the bars, with standard error. Tukey test results that used different letters on bars indicated significant differences at p ≤ 0.05

The spermidine 0.30 mM showed a 34.92% rise paralleled to the control, and 0.45 mM spermidine showed an 41.72% increase at 0KBC levels. When the KBC level was increased to 0.50KBC, the control group showed a root length of 24.15 cm. Adding 0.15 mM spermidine with 0.50KBC treatment led to a modest 7.42% rise contrasted to the control. Increasing the spermidine concentration to 0.30 mM in the 0.50KBC treatment resulted in a notable 20.46% increase evaluated to the control and the highest spermidine concentration in the 0.50KBC treatment, 0.45 mM, indicating a 27.95% increase (Fig. 3A).

In the 0KBC group, 0.15 mM spermidine led to an 8.27% increase in root fresh weight; with 0.30 mM spermidine, a 17.90% increase was observed, and the most remarkable effect occurred at 0.45 mM spermidine, with a 27.06% increase over the control. For the 0.50KBC group, the control had a mean root fresh weight of 28.77 g. The 0.15 mM spermidine with 0.50KBC resulted in a 5.76% rise, while 0.30 mM spermidine showed a 14.72% improvement in root fresh weight in contrast to the control. The most significant effect was recorded at 0.45 mM spermidine with 0.50KBC, which showed a 26.80% increase in root fresh weight than the control (Fig. 3B).

In 0 KBC level, the control group showed a mean root dry weight of 4.91 g. In comparison, when treated with 0.15 mM spermidine at 0KBC level, the root fresh weight increased by 11.11%, and the application of 0.30 mM spermidine resulted in a 26.95% increase. The highest spermidine concentration (0.45 mM) led to a 35.56% increase at 0KBC levels over the control. At 0.50KBC biochar level, the control group showed a root dry weight of 7.71 g. When treated with 0.15 mM spermidine with 0.50KBC, there was an 8.50% increase, and applying 0.30 mM spermidine led to a 15.05% increase in root dry weight (Fig. 3C).

### Number of leaves, leaves fresh and dry weight

**Fig. 4:**
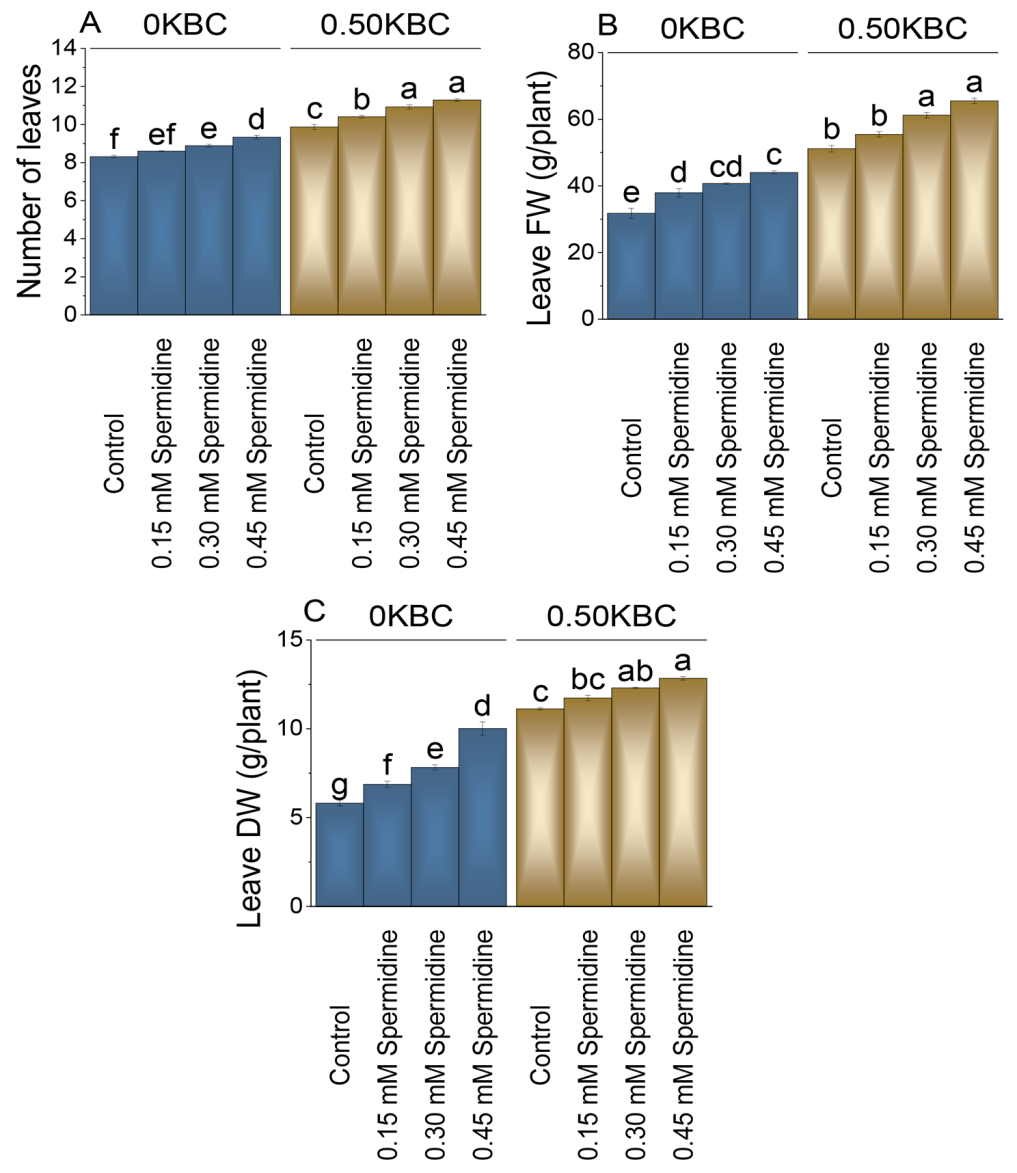
Effect of treatments on number of leaves (**A**), leave fresh weight (**B**), and leave dry weight (**C**) of maize cultivated under 0KBC and 0.50KBC. The means of four replications are represented by the bars, with standard error. Tukey test results that used different letters on bars indicated significant differences at p ≤ 0.05

For the Biochar level 0KBC, the control group had an average number of leaves of 8.31. Adding 0.15 mM spermidine resulted in a modest 3.49% increase, and when the concentration of spermidine was improved to 0.30 mM, there was a more pronounced 6.89% increase in the number of leaves at 0KBC levels over the control. In biochar level 0.50 KBC, the control group had an initial mean of 9.87 leaves. Adding 0.15 mM spermidine led to a 5.47% increase, and the spermidine concentration was increased to 0.30 mM; there was a substantial 10.69% increase in the number of leaves at 0.50KBC contrasted to the control. In comparison, the most remarkable response was 0.45 mM spermidine at 0.50KBC, resulting in a substantial 14.39% increase in leaves number (Fig. 4A).

At 0 KBC level, the application of 0.15 mM spermidine showed a 19.43% increase over the control, and the application of 0.30 mM spermidine exhibited a significant 28.14% rise in leaves fresh weight. In contrast, the 0.45 mM spermidine produced the most significant effect, leading to a remarkable 38.54% increase in leaves fresh weight than the control. When 0.50KBC (0.50% biochar) was applied with 0.15 mM spermidine, it resulted in a modest 8.39% increase in leaves fresh weight, and the application of 0.30 mM spermidine showed a 19.66% increase evaluated to the control. Finally, the highest spermidine concentration (0.45 mM) with 0.50KBC yielded the showed a 28.00% increase from the control (Fig. 4B).

In the 0 KBC group, using 0.15 mM spermidine led to an 18.24% increase in leaves DW, while 0.30 mM spermidine resulted in a more substantial 34.64% increase than the control. The highest increase was observed with 0.45 mM spermidine, where leaves DW exhibited a remarkable 72.42% increase evaluated to the control in the 0KBC treatment. In the 0.50KBC group, a modest 5.49% increase in leaves DW was measured with 0.15 mM spermidine, followed by a 10.59% increase with 0.30 mM spermidine, and a 15.47% increase with 0.45 mM spermidine over the control (Fig. 4C).

### Chlorophyll and carotenoids contents

**Fig. 5:**
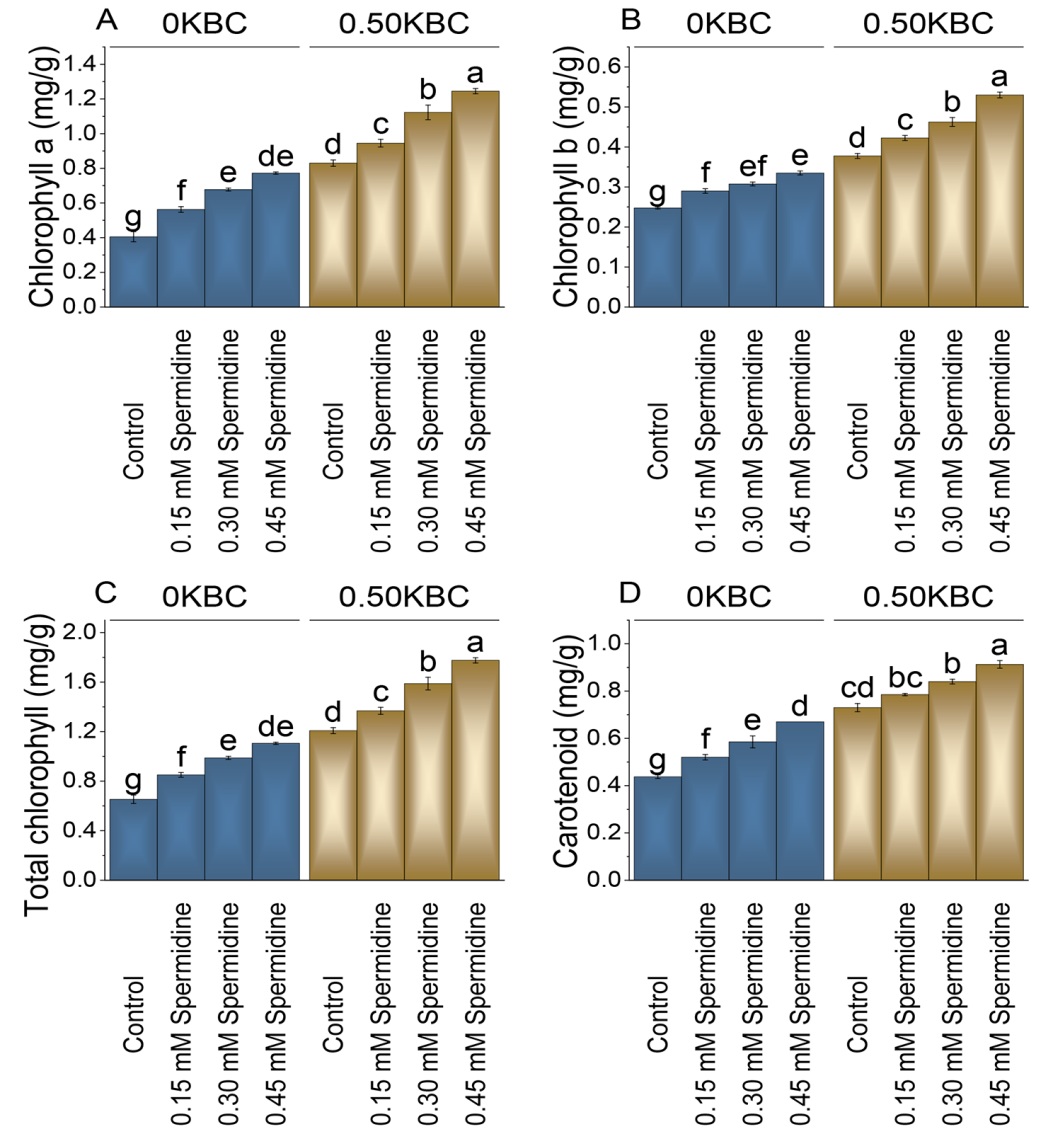
Effect of treatments on chlorophyll a (**A**), chlorophyll b (**B**), total chlorophyll (**C**), and carotenoid (**D**) of maize cultivated under 0KBC and 0.50KBC. The means of four replications are represented by the bars, with standard error. Tukey test results that used different letters on bars indicated significant differences at p ≤ 0.05

The highest concentration of spermidine at 0.45 mM resulted in a remarkable 90.74% increase in chlorophyll content compared to the control. For the 0.50KBC treatment, the control had a baseline chlorophyll a content of 0.83 mg/g. Adding 0.15 mM spermidine led to a 13.86% increase, and when 0.30 mM spermidine was applied, there was a notable 35.24% increase in chlorophyll a content over the control at 0.05 KBC levels (Fig. 5A).

In contrast to the control at 0KBC, when 0.15 mM spermidine treatment was applied, there was a 17.17% increase in Chlorophyll b content; with 0.30 mM spermidine, the Chlorophyll b content increased by 24.24%. The highest increase was observed in the 0.45 mM spermidine treatment at 0KBC group, with a 35.35% rise in Chlorophyll b content evaluated to the control. In the 0.50KBC treatment group contrast to the control, treatment 0.15 mM Spermidine showed an 11.92% increase in Chlorophyll b content and 0.30 mM spermidine, the Chlorophyll b content increased by 22.52%. The most significant increase was observed in the 0.45 mM spermidine treatment group, with a 40.40% rise in Chlorophyll b content in 0.50 KBC (Fig. 5B).

The total chlorophyll content in the 0 KBC control group was 0.65 mg/g, and when treated with 0.15 mM spermidine, it increased by approximately 30.27%. With the application of 0.30 mM spermidine, the total chlorophyll content in the 0KBC group showed a substantial increase of approximately 51.34%, and at 0.45 mM spermidine treatment, the chlorophyll content increased by 69.35% over the control. When subjected to 0.15 mM spermidine, there was a 13.25% increase, and the application of 0.30 mM spermidine in the 0.50KBC group conducted to a significant rise of 31.47% in total chlorophyll content as opposed to the control. The highest increase was observed when 0.45 mM spermidine was used, with a remarkable 47.00% rise in chlorophyll content at 0.50KBC over the control (Fig. 5C).

The carotenoids content in the control group was 0.44 mg/g at 0KBC. When treated with 0.15 mM spermidine, the carotenoids content increased by 18.86%, and the higher spermidine concentration of 0.30 mM resulted in a 33.71% increase in carotenoids content at 0KNC level. The most significant increase was observed at 0.45 mM spermidine at 0KBC, with a 53.14% rise in carotenoids compared to the control. In the 0.50KBC group, the control carotenoids content was 0.73 mg/g. In contrast, adding 0.15 mM spermidine resulted in a 7.53% increase, and when treated with 0.30 mM spermidine at 0.50KBC, carotenoids content increased by 15.07% (Fig. 5D).

### Gass exchange attributes

**Fig. 6:**
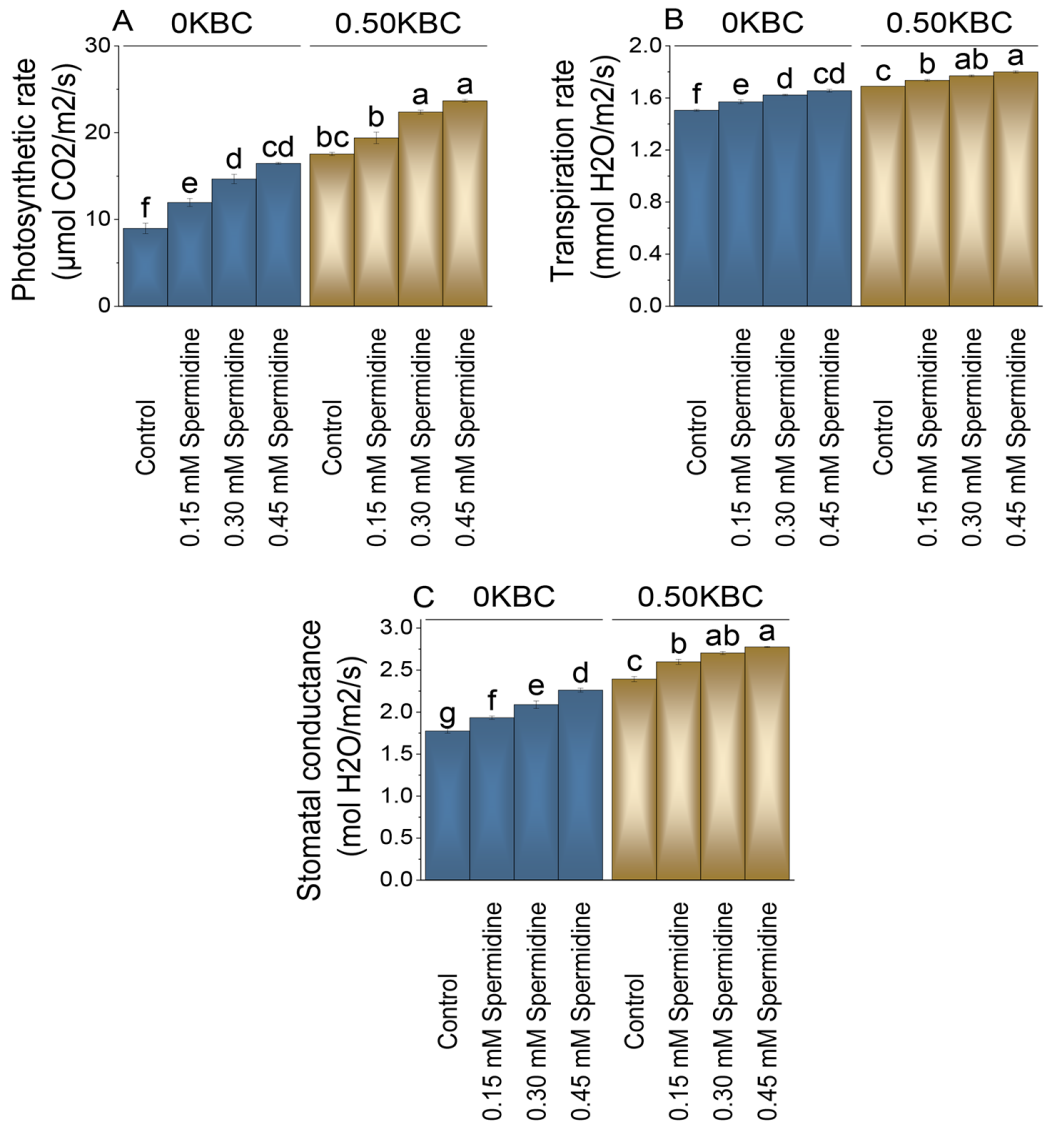
Effect of treatments on photosynthetic rate (**A**), transpiration rate (**B**), and stomatal conductance (**C**) of maize cultivated under 0KBC and 0.50KBC. The means of four replications are represented by the bars, with standard error. Tukey test results that used different letters on bars indicated significant differences at p ≤ 0.05

Without biochar (0KBC), the control group exhibited a photosynthetic rate of 8.98 µmol CO_2_/m^2^/s. In comparison, when treated with 0.15 mM spermidine, the photosynthetic rate increased by 33.23%, and at 0.30 mM spermidine treatment, the photosynthetic rate rose by 63.43%. The highest enhancement in photosynthetic rate within the 0KBC group was observed with 0.45 mM spermidine treatment, which showed an 83.48% rise from the control. The application of 0.30 mM spermidine resulted in a 27.55% increase in photosynthetic rate, and the addition of 0.45 mM spermidine treatment marked a 34.91% increase relative to the control (Fig. 6A).

Under the 0 KBC condition, the control group exhibited a transpiration rate of 1.51 mmol H_2_O/m2/s. When treated with 0.15 mM spermidine at 0 KBC, there was a 4.32% increase in transpiration rate. Applying 0.30 mM spermidine led to a 7.81% increase over the control, and the highest increase in transpiration rate was recorded with 0.45 mM spermidine treatment, which showed a 9.97% increase. In the 0.50KBC treatment group, the control exhibited a transpiration rate of 1.69 mmol H_2_O/m^2^/s. When subjected to 0.15 mM spermidine, the transpiration rate increased by 2.66% compared to the control. Applying 0.30 mM spermidine resulted in a 4.73% increase in transpiration rate, and the highest percentage over the control was measured with 0.45 mM spermidine, which demonstrated a 6.51% increase (Fig. 6B).

In the absence of biochar (0 KBC), the addition of 0.15 mM spermidine resulted in an 8.87% increase in stomatal conductance, which further increased to 17.61% and 27.32% when the spermidine concentration was raised to 0.30 mM and 0.45 mM, respectively in contrast to the control. Conversely, when biochar was introduced at 0.50 KBC, stomatal conductance increased by 8.57% with 0.15 mM spermidine, 12.96% with 0.30 mM spermidine, and 15.99% with 0.45 mM spermidine, in comparison to the control under the 0.50 KBC level in comparison to the control (Fig. 6C).

### Electrolyte leakage, H_2_O_2_, and MDA

**Fig. 7:**
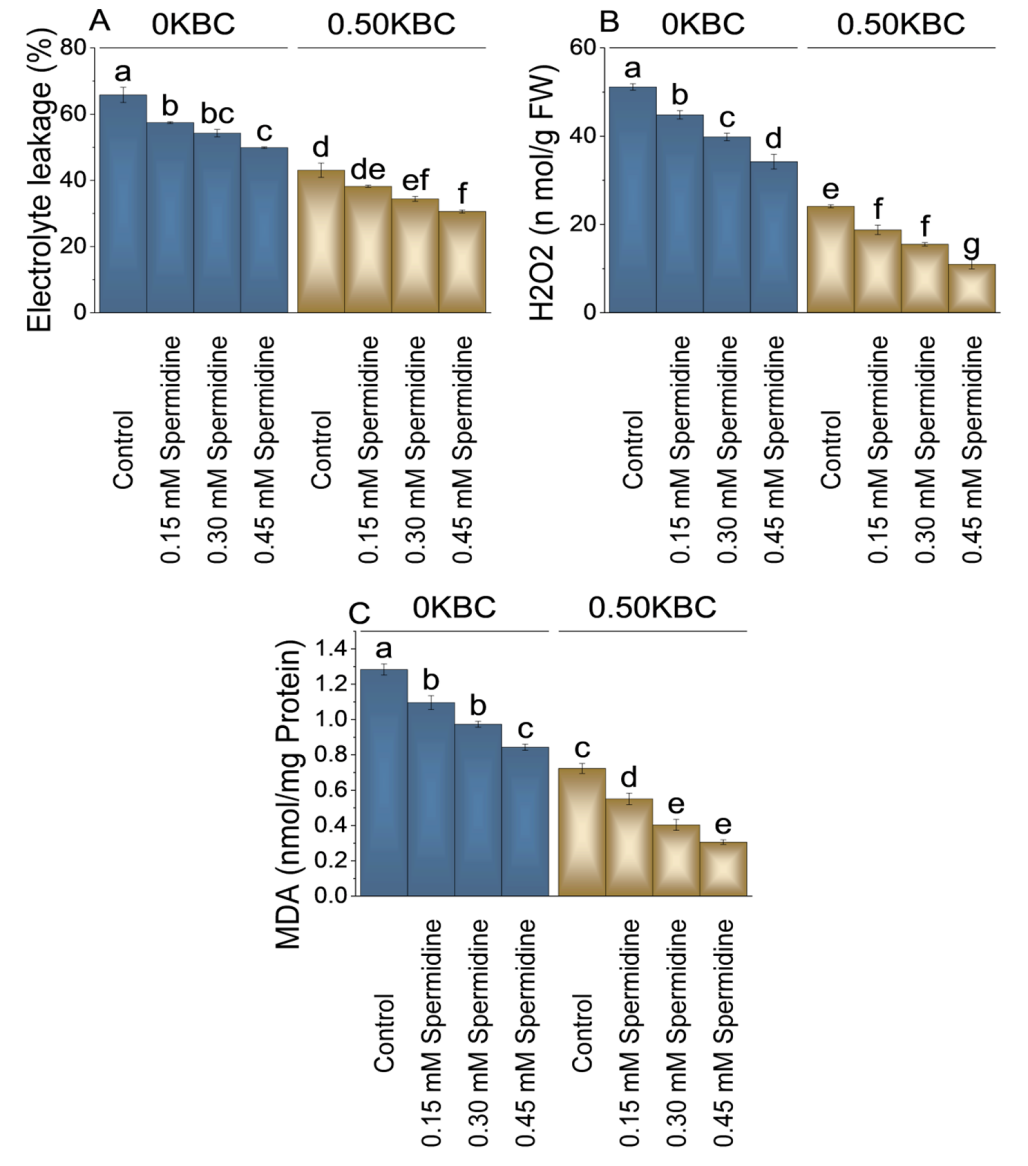
Effect of treatments on electrolyte leakage (**A**), H_2_O_2_ (**B**), and MDA (Malondialdehyde) (**C**) of maize cultivated under 0KBC and 0.50KBC. The means of four replications are represented by the bars, with standard error. Tukey test results that used different letters on bars indicated significant differences at p ≤ 0.05

In the presence of 0.50KBC, the control group exhibited a lower baseline electrolyte leakage of 43.06%. Adding 0.15 mM spermidine resulted in a 12.72% decrease from the control at 0.50KBC. The most significant decrease was observed with 0.45 mM spermidine, showing a remarkable 40.68% reduction in electrolyte leakage than the control (Fig. 7A).

The most significant reduction in H_2_O_2_ levels occurred with 0.45 mM spermidine at 0KBC, showing a 49.48% decrease correlated to the control. At 0.50KBC level, applying 0.15 mM spermidine resulted in a 28.51% reduction in H_2_O_2_ levels, and treatment with 0.30 mM spermidine led to a substantial 55.64% decrease in H_2_O_2_ levels. With 0.45 mM spermidine, an incredible 120.09% reduction in H_2_O_2_ levels was detected within 0.05KBC level compared to the control (Fig. 7B).

The 0.15 mM spermidine resulted in a 17.12% decrease of MDA at 0KBC than the control. Furthermore, when the spermidine concentration was increased to 0.30 mM at 0KBC, a more significant reduction of 31.88% in MDA levels was observed. At 0.45 mM spermidine, there was a remarkable 52.23% decrease in MDA levels related to the control at 0KBC. In the presence of 0.50KBC, adding 0.15 mM spermidine resulted in a 31.36% decrease in MDA levels, with 0.30 mM spermidine resulting in a 79.50% reduction in MDA levels. At 0.45 mM spermidine, the MDA levels showed a remarkable 136.89% decrease at 0.50KBC levels over the control (Fig. 7C).

### POD, SOD, CAT, and APX activity

**Fig. 8:**
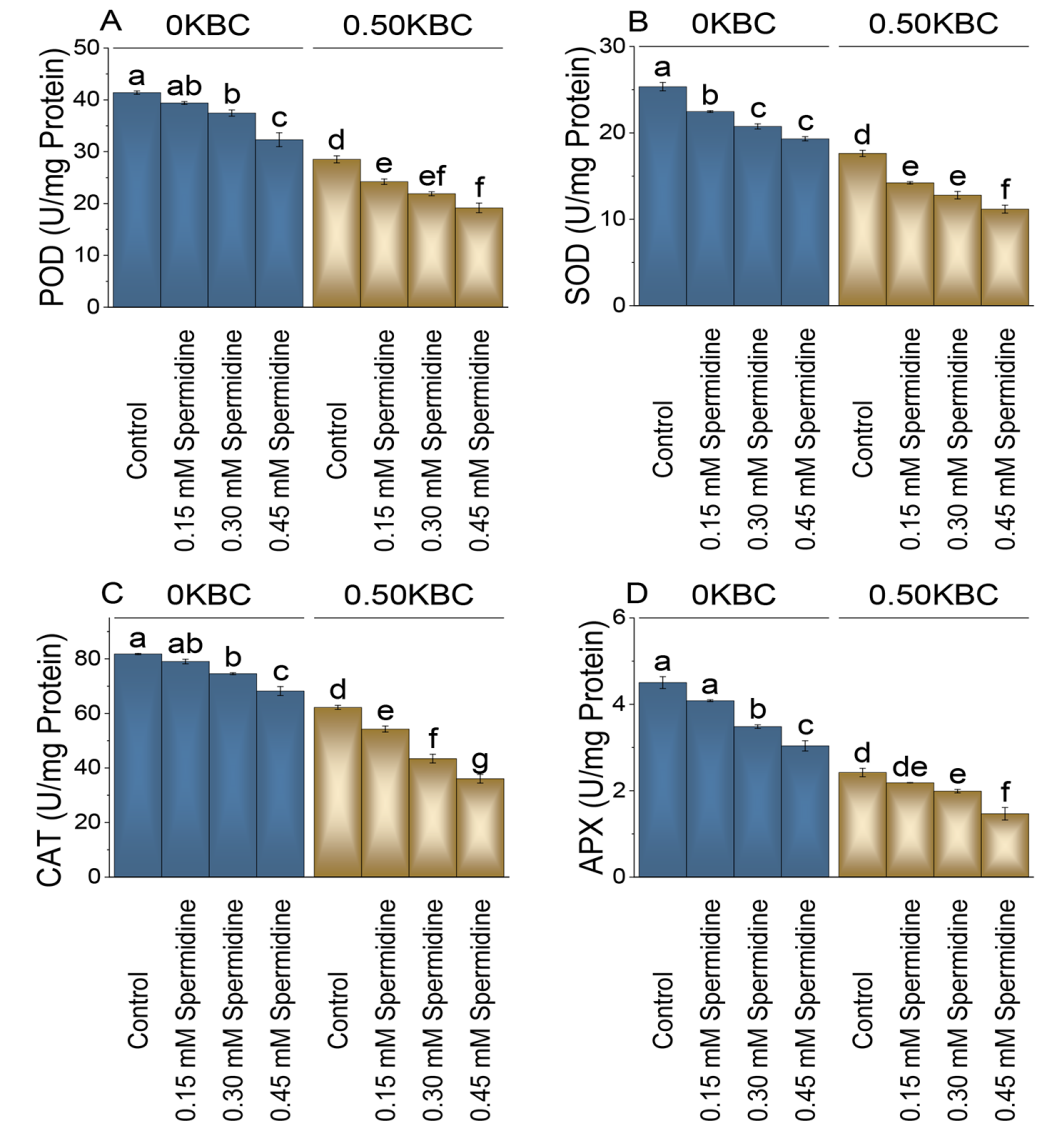
Effect of treatments on POD (Peroxidase) (**A**), SOD (Superoxidase) (**B**), CAT (Catalase) and APX (Ascorbate Peroxidase) (**C**) of maize cultivated under 0KBC and 0.50KBC. The means of four replications are represented by the bars, with standard error. Tukey test results that used different letters on bars indicated significant differences at p ≤ 0.05

The 0.15 mM spermidine at 0KBC, the POD activity decreased by 5.10%-, and 0.30-mM spermidine treatment resulted in a 10.53% reduction in the POD activity. The most significant decrease was observed at 0.45 mM spermidine treatment, where the POD activity dropped by 28.11% compared to the control with 0KBC. In the presence of 0.50% KBC, the control group had a lower POD activity level of 28.52 U/mg Protein. Adding 0.15 mM spermidine resulted in a 17.81% decrease, and a more substantial 30.35% reduction in POD activity was observed with 0.30 mM spermidine over the control. The most pronounced decrease of 48.89% was noted when treated with 0.45 mM spermidine at 0.50KBC evaluated to the control (Fig. 8A).

The SOD (Superoxide Dismutase) enzyme activity was decreased by 12.84% and 22.18% when treated with 0.15 mM and 0.30 mM spermidine evaluated to the control at 0KBC level. The 0.45 mM spermidine treatment led to the most significant reduction in SOD activity, with a 31.25% decrease relative to the control at 0KBC. Treatment of 0.15 mM spermidine with 0.50KBC resulted in a 23.98% decrease in SOD activity, and the application of 0.30 mM spermidine under 0.50KBC conditions further reduced SOD activity by 37.82% (Fig. 8B).

The 0.15 mM spermidine at 0KBC showed a slight decrease of 3.46% in CAT activity. When spermidine concentration was increased to 0.30 mM, there was a more significant reduction in CAT activity, showing a 9.68% decrease compared to the control, and the highest concentration of spermidine (0.45 mM) caused a substantial drop-in CAT activity, with a remarkable 19.91% decrease related to the control. In the case of the 0.50KBC group, treatment 0.15 mM spermidine was added, resulting in a noticeable decrease of 14.59% in CAT activity. The most significant impact on CAT activity was observed with 0.30 mM spermidine, leading to a remarkable 43.24% decrease compared to the control, and the addition of 0.45 mM spermidine in the 0.50KBC group resulted in a substantial 72.52% decrease (Fig. 8C).

In the absence of potassium-rich biochar (0KBC), 0.15 mM spermidine resulted in a 10.29% decrease, 0.30 mM spermidine caused a 29.29% decrease, and 0.45 mM spermidine led to a 48.23% decrease in APX activity over the control. When 0.50KBC was introduced, 0.15 mM spermidine caused a 10.88% decrease, 0.30 mM spermidine led to a 21.76% decrease, and 0.45 mM spermidine resulted in a substantial 65.19% decrease in APX activity contrasted to the control treatment (Fig. 8D).

### Leave nitrogen, phosphorus, and potassium

In the absence of KBC (0KBC), the application of 0.15 mM spermidine resulted in a 3.68% increase, while 0.30 mM spermidine led to a 6.63% increase, and 0.45 mM spermidine led to a 9.30% increase in leave N (%) when compared to the control. When KBC was applied at a concentration of 0.50 KBC, the addition of 0.15 mM spermidine resulted in an 8.05% increase, 0.30 mM spermidine led to an 11.30% increase, and 0.45 mM spermidine resulted in a substantial 13.27% increase in Leave N (%) when related to the control (Table 2).

**Table 2:** Effect of treatment on shoot N, P and K concentration of maize

Spermidine (mM)	Shoot N (%)	Shoot P (%)	Shoot K (%)
	0KBC	0KBC	0KBC
Control	0.14 ± 0.01f	0.08 ± 0.01f	0.29 ± 0.03f
0.15 mM	0.15 ± 0.01ef	0.09 ± 0.01ef	0.44 ± 0.05e
0.30 mM	0.15 ± 0.01de	0.09 ± 0.01de	0.62 ± 0.04d
0.45 mM	0.15 ± 0.01d	0.1 ± 0.01d	0.69 ± 0.03d
	**0.50KBC**	**0.50KBC**	**0.50KBC**
Control	0.16 ± 0.01c	0.11 ± 0.01c	0.91 ± 0.05c
0.15 mM	0.18 ± 0.01b	0.12 ± 0.01c	1.1 ± 0.08fb
0.30 mM	0.18 ± 0.01ab	0.14 ± 0.01ba	1.28 ± 0.06a
0.45 mM	0.19 ± 0.01a	0.17 ± 0.01a	1.37 ± 0.01a

The results are an average of four replicates. At p ≤ 0.05, certain letters revealed significant variations, Tukey Test

At 0 KBC, adding 0.15 mM spermidine caused a 9.06% increase in leave P. In comparison, 0.30 mM spermidine resulted in a more substantial 14.69% increase, and the highest increase was observed with 0.45 mM spermidine, which caused a significant 25.63% rise compared to the control. On the other hand, at 0.50KBC, the application of 0.15 mM spermidine resulted in a 7.73% increase, 0.30 mM spermidine caused a noTable 25.39% increase, and the most substantial increase was observed when 0.45 mM spermidine was applied, leading to a remarkable 48.12% increase related to the control (Table 2).

The application of 0.15 mM spermidine resulted in a 49.44% increase in leave K content over the control in 0KBC. A more substantial increase of 110.76% was observed when 0.30 mM spermidine was applied, and the application of 0.45 mM spermidine yielded the highest increase at 135.78% with 0KBC in contrast to the control. When biochar at the 0.50KBC level was applied, the control group displayed a leave K content of 0.91%. Adding 0.15 mM spermidine resulted in a 20.56% increase in leave K content. A more significant boost of 39.84% was observed with the application of 0.30 mM spermidine, and the highest increase of 49.33% was achieved with 0.45 mM spermidine over the control under 0.50KBC (Table 2).

### Root nitrogen, phosphorus, and potassium

The 0.30 mM spermidine treatment resulted in a more substantial increase of 26.29% in root N, reaching 0.034%, and treatment 0.45 mM spermidine, representing a significant 41.73% increase over the control in the 0KBC level. Moving on to the 0.50KBC treatments, the control group had a root N of 0.040%. The 0.30 mM spermidine treatment for 0.50KBC exhibited an 18.05% increase, and the highest root N% in this group was observed at 0.45 mM spermidine, where the percentage increase was 25.45% evaluated to the control at 0.50KBC level (Table 3).

**Table 3:** Effect of treatment on root N, P and K concentration of maize

Spermidine (mM)	Root N (%)	Root P (%)	Root K (%)
	0KBC	0KBC	0KBC
Control	0.03 ± 0.01e	0.07 ± 0.01f	0.44 ± 0.01f
0.15 mM	0.03 ± 0.01e	0.09 ± 0.01e	0.48 ± 0.01e
0.30 mM	0.03 ± 0.01d	0.12 ± 0.01d	0.54 ± 0.01d
0.45 mM	0.04 ± 0.01c	0.13 ± 0.01d	0.56 ± 0.01 cd
**Spermidine (mM)**	**0.50KBC**	**0.50KBC**	**0.50KBC**
Control	0.04 ± 0.01bc	0.16 ± 0.01c	0.58 ± 0.01c
0.15 mM	0.04 ± 0.01b	0.17 ± 0.01bc	0.63 ± 0.01b
0.30 mM	0.05 ± 0.01a	0.18 ± 0.01b	0.65 ± 0.01b
0.45 mM	0.05 ± 0.01a	0.2 ± 0.01a	0.73 ± 0.02a

The results are an average of four replicates. At p ≤ 0.05, certain letters revealed significant variations, Tukey Test

At 0 KBC, the control had a root P of 0.075, while adding 0.15 mM spermidine led to a 25.08% increase. The 0.30 mM spermidine treatment resulted in a more substantial 59.53% increase, and the highest concentration of spermidine at 0.45 mM caused an even more significant 78.26% increase in contrast to the control. In the presence of 0.50 KBC, the control had a root P of 0.156. Adding 0.15 mM spermidine led to a 9.15% increase in root P over the control, while 0.30 mM spermidine resulted in an 18.46% increase, and 0.45 mM spermidine yielded a 29.70% increase (Table 3).

In 0KBC, the control exhibited a root K of 0.44, while adding 0.15 mM spermidine led to a 10.28% increase. A higher concentration of 0.30 mM spermidine resulted in a 22.27% increase in contrast to the control, and the maximum improvement was observed with 0.45 mM spermidine, yielding a 27.53% increase. When biochar was present at a level of 0.50KBC, the control had a root K of 0.58 (Table 3).

### Convex hull and hierarchical cluster analysis

**Fig. 9:**
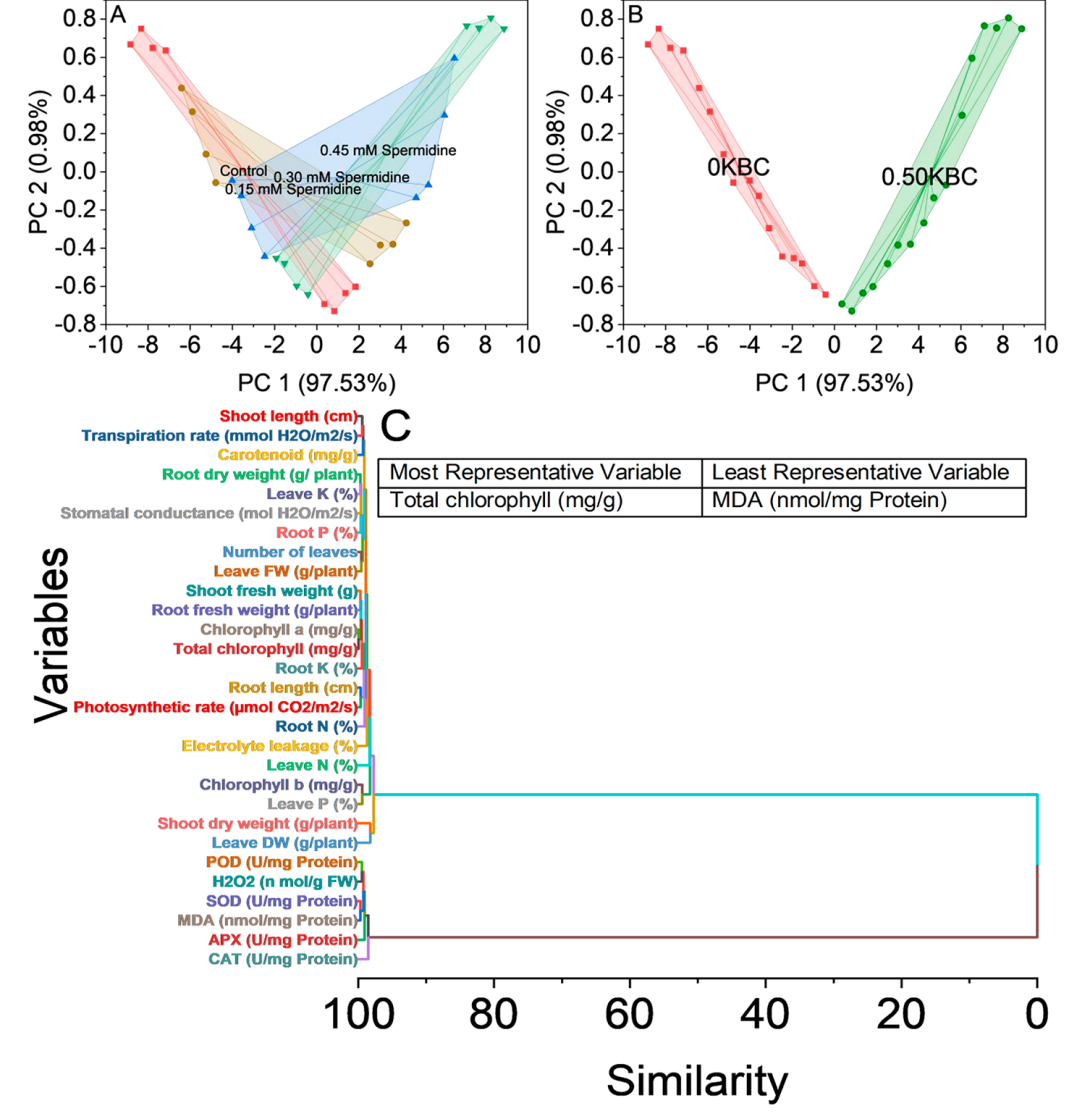
Convex hull cluster plots for treatments (**A**), KBC (**B**), and hierarchical cluster plots for the studied attributes (**C**)

The convex hull analysis assessed the distribution and grouping of data points in a two-dimensional space represented by principal component 1 (PC1) and principal omponent 2 (PC2). The analysis revealed distinct clusters of data points corresponding to different treatments, which provided insights into the treatment effects on the variables under investigation. The data points in the first cluster, labeled as Control, showed a relatively tight grouping, indicating that plants subjected to the control conditions had similar responses. This tight clustering suggested a consistent pattern in the PC1 and PC2 scores for the control treatment. The second cluster, associated with 0.15 mM spermidine treatment, exhibited a dispersion of data points along PC1 and PC2. This dispersion suggested that this treatment led to a more varied response among the samples, with some plants showing distinct responses while others were more like the control group. The third cluster, 0.30 mM spermidine, also showed a spread of data points, implying variability in how plants responded to this specific treatment. The distribution of data points indicated that this treatment had a more diverse effect on the variables represented by PC1 and PC2. The fourth cluster, representing 0.45 mM spermidine treatment, displayed a pattern like the previous clusters. The data points formed a dispersed grouping, signifying varying responses among the samples treated with 0.45 mM spermidine (Fig. 9A).

The convex hull analysis, based on the principal components PC1 and PC2, unveiled distinct groupings of data points associated with different biochar treatments. These results provided valuable insights into how various biochar treatments impacted the plant samples and how they could be categorized. One main cluster, often referred to as the 0KBC Cluster, was characterized by data points displaying a pronounced affinity to the 0KBC biochar treatment. These data points consistently reflected the effects of the 0KBC treatment, as indicated by their PC1 and PC2 scores. Conversely, the second cluster, the 0.50KBC cluster, comprised data points closely associated with the 0.50KBC biochar treatment. Like the first cluster, these data points PC1 and PC2 scores portrayed a distinct response to the 0.50KBC treatment (Fig. 9B).

In the hierarchical cluster analysis conducted in this study, a comprehensive examination of various plant variables was performed to understand their patterns of variation and the relationships between them. The analysis results revealed insights into the similarities and groupings among these variables. First, it was observed that variables associated with chlorophyll content, namely chlorophyll a (mg/g) and total chlorophyll (mg/g), exhibited a high degree of similarity, indicating that they shared common patterns of variation. This suggests a strong relationship between these variables regarding how they respond to different factors or treatments. Furthermore, variables related to plant growth, such as root dry weight (g/plant) and leaves K (%), showed a significant level of similarity. This suggests that changes in these two variables are closely linked and could be influenced by similar factors.

Similarly, shoot fresh weight (g) and root fresh weight (g/plant) were closely related, indicating that they share common patterns in response to varying conditions. Variables associated with oxidative stress, such as SOD (U/mg Protein) and MDA (nmol/mg Protein), exhibited a strong relationship. This suggests that these variables are affected similarly by the presence of oxidative stress-inducing factors. The analysis also unveiled relationships between leaves characteristics and plant performance variables, like stomatal conductance (mol H_2_O/m^2^/s) and root P (%). These variables were found to have shared patterns of variation (Fig. 9C).

### Pearson correlation analysis

**Fig. 10:**
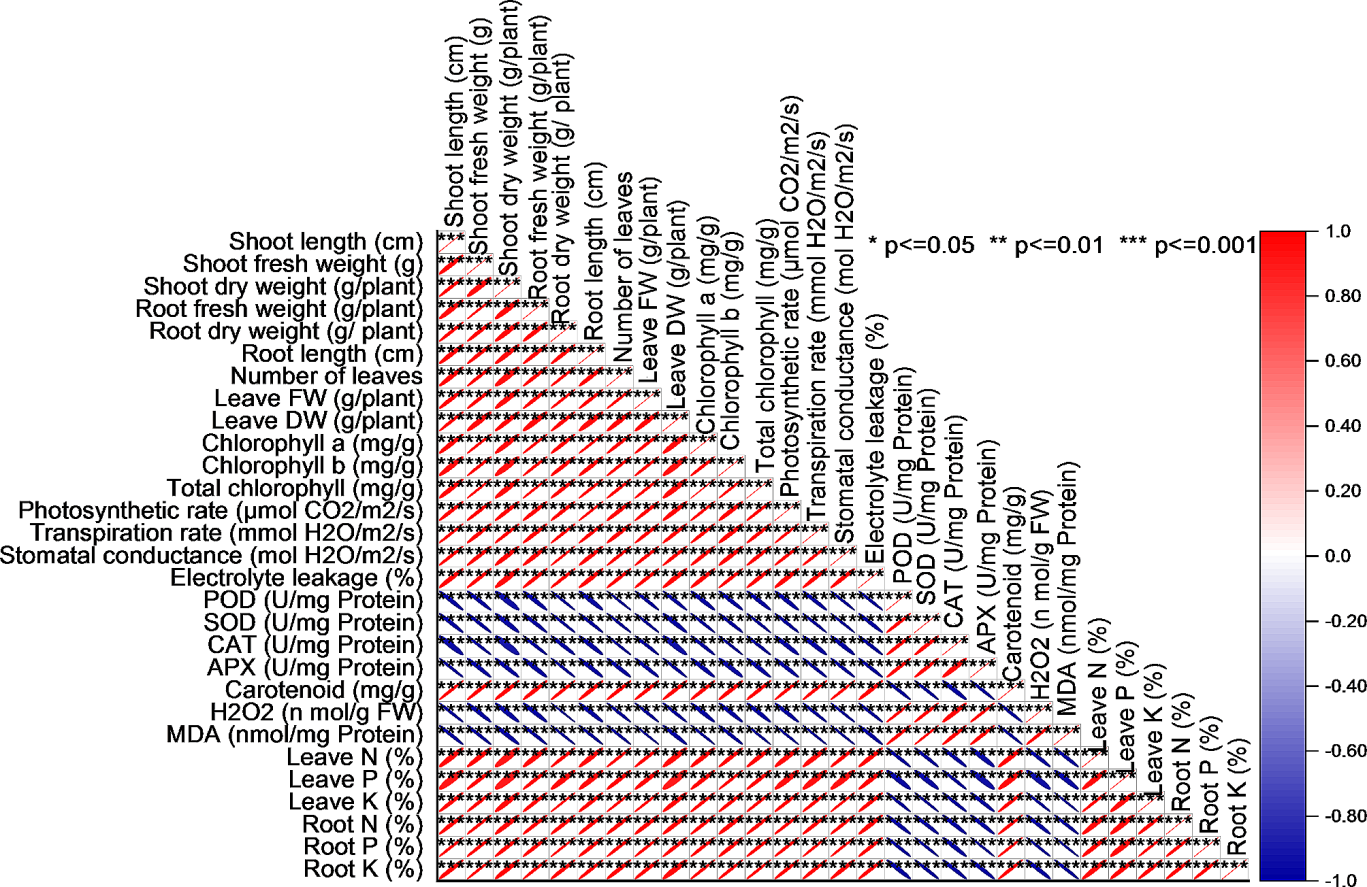
Pearson correlation for studied attributes. The color indicates the intensity of correlation among the parameters

The Pearson correlation analysis conducted in this study aimed to explore the relationships and associations between various plant variables with origin Software. The results revealed several interesting findings regarding the degree of correlation between these variables. First, strong positive correlations were observed between several pairs of variables. Notably, shoot length (cm) exhibited a strong positive correlation (p < 0.05) with shoot fresh weight (g), shoot dry weight (g/plant), root fresh weight (g/plant), root dry weight (g/plant), root length (cm), number of leaves, leaves fresh weight (g/plant), and leaves dry weight (g/plant). These findings suggest that these variables increase or decrease together, indicating vigorous plant growth and characteristics interdependence. Additionally, variables related to photosynthetic activity, such as photosynthetic rate (µmol CO_2_/m^2^/s), transpiration rate (mmol H_2_O/m^2^/s), and stomatal conductance (mol H_2_O/m^2^/s), also displayed strong positive correlations among themselves, further highlighting their interconnectedness. Furthermore, chlorophyll-related variables, namely chlorophyll a (mg/g), chlorophyll b (mg/g), and total chlorophyll (mg/g), exhibited high positive correlations, indicating a consistent pattern in response to certain treatments or conditions. However, it’s worth noting that several variables showed negative correlations, particularly electrolyte leakage (%), POD (U/mg Protein), SOD (U/mg Protein), CAT (U/mg Protein), APX (U/mg Protein), Carotenoid (mg/g), H_2_O_2_ (n mol/g FW), MDA (nmol/mg Protein), leaves N (%), leaves P (%), leave K (%), root N (%), root P (%), and root K (%). These negative correlations suggest that variations in one of these variables are associated with opposite changes in the other (Fig. 10).

## Discussion

Application of spermidine led to elevated concentrations of nitrogen, phosphorus, and potassium in both shoot and root tissues, as compared to the control (Tables 2 and 3). Additionally, spermidine demonstrated heightened values for shoot length, fresh root weight, and shoot dry weight (Fig. 2). This observed enhancement is attributed to spermidine potential to stimulate cell elongation and division, contributing to the elongation of roots and shoots. Existing literature supports the idea that spermidine plays a pivotal role in enhancing nutrient uptake and translocation, particularly in terms of root fresh and dry weight [42]. This enhancement is linked to improved permeability in root cell membranes [43], facilitating nutrient translocation within the plant, ultimately resulting in increased root biomass. Spermidine ability to augment cation exchange capacity in roots proves beneficial for various physiological processes in plants, promoting nutrient uptake by leaves and roots through its influence on root growth, nutrient transporters, and ion exchange [18]. Biochar present in the soil could also help in the enhancement of the nutrient uptake by the maize crop by increasing the water and nutrient holding capacity [44, 45].

Under KBC, spermidine application resulted in an increased number of leaves, leaves fresh weight, and leaves dry weight (Fig. 4). Higher concentrations of spermidine correlated with greater increases in these parameters [46]. This effect is attributed to spermidine’s facilitation of chloroplast development and enhancement of photosynthetic activity, contributing to increased leaves biomass [43]. The elevated dry weight is linked to improved cellular structures, including increased cell wall thickness, driven by spermidine impact on cell division and expansion. Application of biochar increased the impact of spermidine on leave number and fresh weight, which might be due to improvement in soil health conditions and availability of water conditions [47, 48].

Further observations revealed increased chlorophyll a, b, and total content, photosynthetic rate, transpiration, and stomatal conductance with spermidine application under biochar conditions (Figs. 5 and 6). The concentration-dependent increase in these parameters is attributed to spermidine ability to enhance photosynthesis through improved chloroplast development and efficiency [49]. Spermidine plays a protective role in stress responses by stabilizing photosynthetic pigments and stimulating chlorophyll synthesis, ultimately contributing to increased light absorption and enhanced photosynthesis [50].

Electrolyte leakage, peroxidase, superoxide dismutase, catalase, and ascorbate peroxidase levels were higher in the control group without spermidine application, and even higher values were observed in the absence of biochar (Figs. 8 and 9). Spermidine’s impact on these parameters indicates its role in maintaining membrane integrity, quenching reactive oxygen species, and reducing oxidative stress [51]. The concentration-dependent effects of spermidine on antioxidant enzyme activity highlight a delicate balance between reactive oxygen species scavenging and production regulation, crucial for optimizing antioxidant function without inducing oxidative stress. In addition, the proposition that the application of biochar can enhance water availability and uptake by porous structure inherent in biochar. The inherent porosity and notable water-holding capacity of biochar have the potential to impact soil water dynamics positively. The porous structure enables biochar to function as a reservoir for water, mitigating drainage and promoting increased water retention in the soil [52]. Consequently, this structural characteristic of biochar may contribute to elevated water availability for plant uptake [53], concurrently leading to reduced production of antioxidants in conditions where biochar is applied. The application of biochar to various soils enhanced the presence of proteobacteria. Proteobacteria encompass most ammonia-oxidizing bacteria, including nitrogen-fixing bacteria, ammonia-oxidizing bacteria, cellulose-decomposing bacteria, nitrifying bacteria, and denitrifying bacteria. This microbial group plays a crucial role in nitrogen recycling, contributing to the overall well-being of plants by positively influencing growth, yield, and the quality of fruits and seeds [54].

## Conclusion

In conclusion, the utilization of 0.45 mM spermidine in conjunction with 0.50% KBC presents a promising strategy to enhance maize growth. The combined application of 0.45 mM spermidine with 0.50% KBC demonstrates the capability to augment both root and shoot nutrient uptake, especially in essential elements such as phosphorus (P), nitrogen (N), and potassium (K), crucial for fostering robust maize growth. Moreover, the treatment involving 0.45 mM spermidine with 0.50% KBC displays effective regulation of antioxidant mechanisms. To assess the effectiveness of the 0.45 mM spermidine treatment and 0.50KBC as an optimal solution for promoting maize growth.

## Data Availability

All data generated or analysed during this study are included in this published article.

## References

[1] Salika R, Riffat J (2021). Abiotic stress responses in maize: a review. Acta Physiol Plant.

[2] Mohamed Salem H, Ali AM (2023). Effect of Olive Mill Wastes on Soil Physicochemical properties and Maize Yield under saline soil conditions. J Soil Plant Environ.

[3] Choudhary VK. Weed suppression, weed seed bank and crop productivity influenced under tillage and mulches in maize-rapeseed cropping system. Crop Prot. 2023;106333.

[4] Zingore S. Integrated soil fertility management: a basis for sustainable intensification of maize-based cropping systems of Southern Africa. In: Zingore S, editor. Towards sustainable food production in africa: best management practices and technologies. Singapore: Springer Nature Singapore; 2023. p. 39–57.

[5] FAO. National policy dialogue on salt-affected soils. 2017. https://www.fao.org/pakistan/news/detail-events/zh/c/522681/.

[6] Bhusal B, Poudel MR, Rishav P, Regmi R, Neupane P, Bhattarai K (2021). A review on abiotic stress resistance in maize (Zea mays L.): effects, resistance mechanisms and management. J Biol Today’s World.

[7] Nawaz H (2023). Pigeon Pea Green Manuring and Nitrogen fertilization increase agronomic efficiency by improving yield and ear characteristics of Maize. J Soil Plant Environ.

[8] Younis U, Qayyum MF, Shah MHR, Danish S, Shahzad AN, Malik SA, et al. Growth, survival, and heavy metal (Cd and Ni) uptake of spinach (*Spinacia oleracea*) and fenugreek (*Trigonella corniculata*) in a biochar-amended sewage-irrigated contaminated soil. J Plant Nutr Soil Sci. 2015;178.

[9] Alotaibi MO, Ikram M, Alotaibi NM, Hussain GS, Ghoneim AM, Younis U (2023). Examining the role of AMF-Biochar in the regulation of spinach growth attributes, nutrients concentrations, and antioxidant enzymes in mitigating drought stress. Plant Stress.

[10] Nawaz A, Tariq M, Khan K, Haq MU, Khan H (2023). Integrated Effect of Heavy Metal-Tolerant Rhizobacteria and Phosphorus on Maize Growth and Phosphorus Bioavailability in Contaminated Soil. J Soil Plant Environ.

[11] Mamarasulov B, Davranov K, Jahan MS, Jabborova D, Nasif O, Ansari MJ et al. Characterization, enzymatic and biochemical properties of endophytic bacterial strains of the medicinal plant *Ajuga turkestanica* (Rgl.) Brig (Lamiaceae). J King Saud Univ Sci. 2022;34:102183.

[12] Sheikh L, Younis U, Shahzad AS, Hareem M, Noor Elahi N, Danish S (2023). Evaluating the effects of cadmium under saline conditions on leafy vegetables by using acidified biochar. Pak J Bot.

[13] Shahzad AS, Younis U, Naz N, Danish S, Syed A, Elgorban AM (2023). Acidified biochar improves lead tolerance and enhances morphological and biochemical attributes of mint in saline soil. Sci Rep.

[14] Anwar T, Shehzadi A, Qureshi H, Shah MN, Danish S, Salmen SH (2023). Alleviation of cadmium and drought stress in wheat by improving growth and chlorophyll contents amended with GA3 enriched deashed biochar. Sci Rep.

[15] Bilias F, Kalderis D, Richardson C, Barbayiannis N, Gasparatos D (2023). Biochar application as a soil potassium management strategy: a review. Sci Total Environ.

[16] Dai Y, Zheng H, Jiang Z, Xing B. Combined effects of biochar properties and soil conditions on plant growth: a meta-analysis. Sci Total Environ. 2020;713:136635. 10.1016/j.scitotenv.2020.136635 32019022

[17] Purakayastha TJ, Bera T, Bhaduri D, Sarkar B, Mandal S, Wade P (2019). A review on biochar modulated soil condition improvements and nutrient dynamics concerning crop yields: pathways to climate change mitigation and global food security. Chemosphere.

[18] Liu C, Lan C, Li C, Li C, Huang J (2023). Exogenous spermidine and calcium alleviate waterlogging stress in cherry tomato at the seedling stage. Sci Hortic.

[19] Alamer KH (2023). Combined effect of trehalose and spermidine to alleviate zinc toxicity in Vigna radiata. 3 Biotech.

[20] Ahanger MA, Aziz U, Alsahli A, Alyemeni MN, Ahmad P (2020). Combined kinetin and spermidine treatments ameliorate growth and photosynthetic inhibition in Vigna angularis by up-regulating antioxidant and nitrogen metabolism under cadmium stress. Biomolecules.

[21] Gee GW, Bauder JW. Particle-size analysis. In: Methods of soil analysis. Part 1. Physical and mineralogical methods. 2nd edition. Madison; 1986. p. 383–411.

[22] McLean EO. Soil pH and lime requirement. In: Page AL, editor. Methods of Soil Analysis: Part 2 Chemical and Microbiological Properties. 2nd edition. American Society of Agronomy, Crop Science Society of America, and Soil Science Society of America; 1982. p. 199–224.

[23] Rhoades JD, Salinity. Electrical Conductivity and Total Dissolved Solids. Methods of Soil Analysis, Part 3: Chemical Methods. 2018;:417–35.

[24] Walkley A (1935). An examination of methods for determining Organic Carbon and Nitrogen in Soils. J Agric Sci.

[25] Bremner JM, Mulvaney CS, Page AL, Miller RH, Keeney DR (1982). Nitrogen–total. Methods of soil analysis. Part 2. Chemical and microbiological properties.

[26] Kuo S, Sparks DL, Page AL, Helmke PA, Loeppert RH, Soltanpour PN, Tabatabai MA (2018). Phosphorus. Methods of Soil Analysis Part 3: Chemical methods.

[27] Pratt PF. Potassium. In: Norman AG, editor. Methods of Soil Analysis, Part 2: Chemical and Microbiological properties. John Wiley & Sons, Ltd; 2016. pp. 1022–30.

[28] Keeney DR, Nelson DW. Nitrogen-inorganic forms. In: Page AL, editor. Methods of Soil Analysis: Part 2 Chemical and Microbiological Properties, 9.2.2. 2nd edition. American Society of Agronomy, Crop Science Society of America, and Soil Science Society of America; 1983. p. 643–98.

[29] Rhoades JD, Sparks DL, Page AL, Helmke PA, Loeppert RH, Soltanpour PN, Tabatabai MA (1996). Salinity: electrical conductivity and total dissolved solids. Methods of Soil Analysis, Part 3, Chemical methods.

[30] V. J. C. H. Schouwvenberg and I. Walinge. Methods of analysis for Plant Material., Agriculture University, Wageningen. 1973.

[31] Miller R. Nitric-perchloric Acid Wet Digestion In An Open Vessel. In: Kalra Y, editor. Handbook of Reference Methods for Plant Analysis. 1st edition. Washington, D.C.: CRC Press; 1997. p. 57–62.

[32] Ahmad M, Rajapaksha AU, Lim JE, Zhang M, Bolan N, Mohan D (2014). Biochar as a sorbent for contaminant management in soil and water: a review. Chemosphere.

[33] Arnon DI (1949). Copper enzymes in isolated chloroplasts. Polyphenoloxidase in Beta vulgaris. Plant Physiol.

[34] Nazar R, Khan MIR, Iqbal N, Masood A, Khan NA (2014). Involvement of ethylene in reversal of salt-inhibited photosynthesis by sulfur in mustard. Physiol Plant.

[35] Dhindsa RS, Plumb-Dhindsa PL, Reid DM (1982). Leaf senescence and lipid peroxidation: effects of some phytohormones, and scavengers of free radicals and singlet oxygen. Physiol Plant.

[36] Hori M, Kondo H, Ariyoshi N, Yamada H, Hiratsuka A, Watabe T (1997). Changes in the hepatic glutathione peroxidase redox system produced by coplanar polychlorinated biphenyls in Ah-responsive and-less-responsive strains of mice: mechanism and implications for toxicity. Environ Toxicol Pharmacol.

[37] Aebi H. Catalase in vitro. Methods in Enzymology. Academic Press Inc.; 1984. pp. 121–6. 10.1016/s0076-6879(84)05016-3 6727660

[38] Nakano Y, Asada K (1981). Hydrogen peroxide is scavenged by ascorbate-specific peroxidase in spinach chloroplasts. Plant Cell Physiol.

[39] Lutts S, Kinet JM, Bouharmont J (1996). NaCl-induced senescence in leaves of rice (Oryza sativa L.) cultivars differing in salinity resistance. Ann Bot.

[40] Steyermark AL, McGee BE (1961). Progress in elemental quantitative organic analysis: 1960. Microchem J.

[41] Olsen SR, Sommers LE, Page AL, editors. others. Methods of soil analysis. Part. 1982;2:403–30.

[42] Salah A, Nwafor CC, Han Y, Liu L, Rashid M, Batool M (2022). Spermidine and brassinosteroid regulate root anatomical structure, photosynthetic traits and antioxidant defense systems to alleviate waterlogging stress in maize seedlings. South Afr J Bot.

[43] Amri E, Shahsavar AR (2010). Response of Lime Seedlings (Citrus aurantifolia L.) to exogenous spermidine treatments under Drought stress. Aust J Basic Appl Sci.

[44] Gul S, Whalen JK (2016). Biochemical cycling of nitrogen and phosphorus in biochar-amended soils. Soil Biol Biochem.

[45] Mandal S, Thangarajan R, Bolan NS, Sarkar B, Khan N, Ok YS (2016). Biochar-induced concomitant decrease in ammonia volatilization and increase in nitrogen use efficiency by wheat. Chemosphere.

[46] Nandy S, Das T, Tudu CK, Mishra T, Ghorai M, Gadekar VS (2022). Unravelling the multi-faceted regulatory role of polyamines in plant biotechnology, transgenics and secondary metabolomics. Appl Microbiol Biotechnol.

[47] Agegnehu G, Srivastava AK, Bird MI (2017). The role of biochar and biochar-compost in improving soil quality and crop performance: a review. Appl Soil Ecol.

[48] Chen S, Yang M, Ba C, Yu S, Jiang Y, Zou H (2018). Preparation and characterization of slow-release fertilizer encapsulated by biochar-based waterborne copolymers. Sci Total Environ.

[49] Shi Y, Zhao Y, Yao Q, Liu F, Li X, Jin X (2022). Comparative physiological and transcriptomic analyses reveal mechanisms of Exogenous Spermidine-Induced Tolerance to low-Iron stress in Solanum lycopersicum L. Antioxidants.

[50] Yang X, Han Y, Hao J, Qin X, Liu C, Fan S (2022). Exogenous spermidine enhances the photosynthesis and ultrastructure of lettuce seedlings under high-temperature stress. Sci Hortic.

[51] Bahari Saravi H, Gholami A, Pirdashti H, Baradaran Firouzabadi M, Asghari H, Yaghoubian Y (2022). Improvement of salt tolerance in Stevia rebaudiana by co-application of endophytic fungi and exogenous spermidine. Ind Crops Prod.

[52] Liu Z, Dugan B, Masiello CA, Gonnermann HM. Biochar particle size, shape, and porosity act together to influence soil water properties. PLoS ONE. 2017;12. 10.1371/journal.pone.0179079. 10.1371/journal.pone.0179079 PMC5466324 28598988

[53] Suliman W, Harsh JB, Abu-lail NI, Fortuna A, Dallmeyer I, Garcia-pérezassociate M (2017). The role of biochar porosity and surface functionality in augmenting hydrologic properties of a sandy soil. Sci Total Environ the.

[54] Zhang H, Ullah F, Ahmad R, Ali Shah SU, Khan A, Adnan M (2022). Response of soil proteobacteria to biochar amendment in sustainable agriculture- A mini review. J Soil Plant Environ.

